# Correction: Validation of the Mentalization Scale (MentS) in francophone control and clinical samples

**DOI:** 10.1371/journal.pone.0342173

**Published:** 2026-02-02

**Authors:** The PLOS One Staff

There are errors in the author affiliations. The correct affiliations are as follows:

Flora Descartes^1^, Vincent Besch^1^, Margaux Bouteloup^1,5^, Rosetta Nicastro^3,4^, Eléonore Pham^3,4^, Eva Rüfenacht^3,4^, Nader Ali Perroud^3,4,^ Martin Debbané^1,2^

**1** Developmental Clinical Psychology Research Unit, Faculty of Psychology and Educational Sciences, University of Geneva, Switzerland, **2** Research Department of Clinical, Educational and Health, Psychology, University College London, United Kingdom, **3** Faculty of Medicine, Geneva University, Geneva, Switzerland, **4** Department of Psychiatry, Geneva University Hospitals, Geneva, Switzerland, 5 Université Marie et Louis Pasteur, UFR SLHS, Besançon, France

The publisher apologizes for the errors.

## References

[1] Descartes F, Besch V, Bouteloup M, Nicastro R, Pham E, Rüfenacht E, et al. Validation of the Mentalization Scale (MentS) in francophone control and clinical samples. PLoS One. 2025;20(10):e0332724. doi: 10.1371/journal.pone.0332724 41150688 PMC12561985

