# Supplementary material for: Validation of the Mentalization Scale (MentS) in francophone control and clinical samples
Source: PLoS One. 2025 Oct 28;20(10):e0332724. doi: 10.1371/journal.pone.0332724 (PMC12561985; doi:10.1371/journal.pone.0332724)
Supplement: S1 Data — S1 Table. Full Table 4 Correlations in control sample - Spearman’s rho Correlations. S2 Table. Full Table 5 Correlations in clinical sample - Spearman’s rho Correlations. S3 File. French translation of The Mentalization Scale (MentS). S4 List of researchers who contributed to this work as part of the RF-TBM Consortium. (DOCX) [file pone.0332724.s001.docx]

**Supplementary material**

**S1 Table. Full Table 5 Correlations in control sample-Spearman's rho Correlations.**

|  | | | **MentS_Total** | **MentS_M** | **MentS_O** | **MentS_S** | **SCL90_GSI** | **CERQ_Ad_ER** | **RFQ_**  **c** | **CTQ_**  **EmAb** | **CTQ_PhAb** | **CTQ_**  **SexAb** | **CTQ_**  **EmNeg** | **CTQ_**  **PhNeg** | **CTQ_Tot** |
| --- | --- | --- | --- | --- | --- | --- | --- | --- | --- | --- | --- | --- | --- | --- | --- |
|  | **MentS_Tot** |  | -- |  |  |  |  |  |  |  |  |  |  |  |  |
|  | **MentS_M** |  | .793^**^ | -- |  |  |  |  |  |  |  |  |  |  |  |
|  | **MentS_O** |  | .785^**^ | .703^**^ | -- |  |  |  |  |  |  |  |  |  |  |
|  | **MentS_S** |  | .603^**^ | .120^*^ | .175^**^ | -- |  |  |  |  |  |  |  |  |  |
|  | **SCL90_GSI** |  | -.164^**^ | .111 | .078 | -.520^**^ | -- |  |  |  |  |  |  |  |  |
|  | **CERQ_Ad_ER** |  | .282^**^ | .183^**^ | .184^**^ | .254^**^ | -.270^**^ | -- |  |  |  |  |  |  |  |
|  | **RFQ_c** |  | .418^**^ | .117^*^ | .218^**^ | .602^**^ | -.496^**^ | .233^**^ | -- |  |  |  |  |  |  |
|  | **CTQ_EmAb** |  | .020 | .161^**^ | .161^**^ | -.221^**^ | .401^**^ | -.069 | -.175^**^ | -- |  |  |  |  |  |
|  | **CTQ_PhAb** |  | .035 | .062 | .057 | -.052 | .166^**^ | .018 | -.126^*^ | .555^**^ | -- |  |  |  |  |
|  | **CTQ_SeAb** |  | .042 | .124^*^ | .100 | -.120^*^ | .150^**^ | .004 | -.126^*^ | .343^**^ | .358^**^ | -- |  |  |  |
|  | **CTQ_EmNeg** |  | -.072 | .008 | .028 | -.149^**^ | .270^**^ | -.181^**^ | -.103 | .664^**^ | .449^**^ | .219^**^ | -- |  |  |
|  | **CTQ_PhNeg** |  | -.144^*^ | -.068 | -.089 | -.156^**^ | .218^**^ | -.083 | -.197^**^ | .417^**^ | .367^**^ | .252^**^ | .574^**^ | -- |  |
|  | **CTQ_Tot** |  | -.045 | .074 | .070 | -.201^**^ | .354^**^ | -.120^*^ | -.180^**^ | .844^**^ | .617^**^ | .407^**^ | .901^**^ | .690^**^ | -- |
| **. Correlation is significant at the 0.01 level (2-tailed).  *. Correlation is significant at the 0.05 level (2-tailed). | | | | | | | | | | | | | | |  |

**S2 Table. Full Table 6 Correlations in clinical sample – Spearman's rho Correlations.**

|  | | MentS_Tot | MentS_M | MentS_O | MentS_  S | BSL_  23 | CERQ_Ad_ER | RFQ_c | CTQ_  EmAb | CTQ_  Phab | CTQ-SexAb | CTQ_EmNeg | CTQ_  PhNeg | CTQ_  Tot | |
| --- | --- | --- | --- | --- | --- | --- | --- | --- | --- | --- | --- | --- | --- | --- | --- |
|  | MentS_Tot | -- |  |  |  |  |  |  |  |  |  |  |  |  | |
|  | MentS_M | .856^**^ | -- |  |  |  |  |  |  |  |  |  |  |  | |
|  | MentS_O | .762^**^ | .623^**^ | -- |  |  |  |  |  |  |  |  |  |  | |
|  | MentS_S | .673^**^ | .359^**^ | .209^**^ | -- |  |  |  |  |  |  |  |  |  | |
|  | BSL_23 | -.145^**^ | .005 | .085 | -.425^**^ | -- |  |  |  |  |  |  |  |  | |
|  | CERQ_Ad_ER | .265^**^ | .208^**^ | .112^*^ | .294^**^ | -.405^**^ | -- |  |  |  |  |  |  |  | |
|  | RFQ_c | .431^**^ | .237^**^ | .266^**^ | .521^**^ | -.332^**^ | .253^**^ | -- |  |  |  |  |  |  | |
|  | CTQ_EmAb | .033 | .122^*^ | .144^**^ | -.183^**^ | .435^**^ | -.198^**^ | -.168^**^ | -- |  |  |  |  |  | |
|  | CTQ_PhAb | -.039 | -.018 | .046 | -.128^**^ | .197^**^ | -.115^*^ | -.105^*^ | .566^**^ | -- |  |  |  |  | |
|  | CTQ_SexAb | .066 | .110^*^ | .053 | -.036 | .236^**^ | -.053 | -.066 | .391^**^ | .281^**^ | -- |  |  |  | |
|  | CTQ_EmNeg | -.099^*^ | -.014 | .000 | -.206^**^ | .349^**^ | -.177^**^ | -.178^**^ | .693^**^ | .488^**^ | .336^**^ | -- |  |  | |
|  | CTQ_PhNeg | -.145^**^ | -.092 | -.013 | -.206^**^ | .290^**^ | -.115^*^ | -.176^**^ | .520^**^ | .439^**^ | .285^**^ | .645^**^ | -- |  | |
|  | CTQ_Tot | -.026 | .060 | .082 | -.199^**^ | .419^**^ | -.175^**^ | -.189^**^ | .881^**^ | .661^**^ | .564^**^ | .861^**^ | .712^**^ | -- | |
| **. Correlation is significant at the 0.01 level (2-tailed).  *. Correlation is significant at the 0.05 level (2-tailed). | | | | | | | | | | | | | | |  |

**S3. The Mentalization Scale (MentS) French translation**

Ce questionnaire porte sur la façon dont vous réfléchissez à vos émotions, pensées et actions, ainsi qu’à celles des autres. Lisez attentivement chaque énoncé et indiquez votre degré d’accord dans la colonne de droite.

|  |  | ***Totalement faux*** | ***Modérément faux*** | ***À la fois vrai et faux*** | ***Modérément vrai*** | ***Totalement vrai*** |
| --- | --- | --- | --- | --- | --- | --- |
| 1 | Je trouve important de comprendre les raisons de mon comportement. | 1 | 2 | 3 | 4 | 5 |
| 2 | J’observe soigneusement ce que les autres disent et font lorsque j’émets des conclusions quant à leurs traits de personnalité. | 1 | 2 | 3 | 4 | 5 |
| 3 | Je peux reconnaître les sentiments des autres. | 1 | 2 | 3 | 4 | 5 |
| 4 | Je pense souvent aux autres et à leur comportement. | 1 | 2 | 3 | 4 | 5 |
| 5 | Je peux généralement identifier ce qui rend les autres inquiets. | 1 | 2 | 3 | 4 | 5 |
| 6 | Je peux compatir avec les sentiments des autres. | 1 | 2 | 3 | 4 | 5 |
| 7 | Lorsque quelqu’un m’agace, j’essaie de comprendre pourquoi je réagis de cette façon. | 1 | 2 | 3 | 4 | 5 |
| 8 | Lorsque je suis contrarié(e), je suis incertain(e) si je suis triste, si j’ai peur ou si je suis en colère. | 1 | 2 | 3 | 4 | 5 |
| 9 | Je n’aime pas perdre mon temps à essayer de comprendre en détail le comportement des autres. | 1 | 2 | 3 | 4 | 5 |
| 10 | Je peux faire de bonnes prédictions du comportement des autres lorsque je connais leurs croyances et leurs sentiments. | 1 | 2 | 3 | 4 | 5 |
| 11 | Souvent, je ne peux pas expliquer, même à moi-même, pourquoi j’ai agi d’une certaine façon. | 1 | 2 | 3 | 4 | 5 |
| 12 | Parfois, je peux comprendre le sentiment qu’éprouve quelqu’un d’autre avant qu’il/elle me le dise. | 1 | 2 | 3 | 4 | 5 |
| 13 | Je trouve qu’il est important de comprendre ce qui se passe dans ma relation avec les gens dont je suis proche. | 1 | 2 | 3 | 4 | 5 |
| 14 | Je ne veux pas découvrir quelque chose à propos de moi-même que je n’aimerais pas. | 1 | 2 | 3 | 4 | 5 |
| 15 | Il faut connaître les pensées, les désirs et les sentiments de quelqu’un d’autre pour comprendre son comportement. | 1 | 2 | 3 | 4 | 5 |
| 16 | Je parle fréquemment des émotions avec les gens dont je suis proche. | 1 | 2 | 3 | 4 | 5 |
| 17 | J’aime lire des livres et des articles de journaux portant sur des sujets reliés à la psychologie. | 1 | 2 | 3 | 4 | 5 |
| 18 | Je trouve difficile de m’avouer quand je suis triste, blessé(e) ou que j’ai peur. | 1 | 2 | 3 | 4 | 5 |
| 19 | Je n’aime pas penser à mes problèmes. | 1 | 2 | 3 | 4 | 5 |
| 20 | Je peux décrire en précision et avec détails des traits significatifs chez les gens dont je suis proche. | 1 | 2 | 3 | 4 | 5 |
| 21 | Je suis souvent confus vis-à-vis mes sentiments exacts. | 1 | 2 | 3 | 4 | 5 |
| 22 | Il m’est difficile de trouver les mots adéquats pour décrire mes sentiments. | 1 | 2 | 3 | 4 | 5 |
| 23 | Les gens me disent que je les comprends et que je leur donne de bons conseils. | 1 | 2 | 3 | 4 | 5 |
| 24 | Je me suis toujours intéressé(e) aux raisons pour lesquelles les gens agissaient de certaines manières. | 1 | 2 | 3 | 4 | 5 |
| 25 | Je peux facilement décrire ce que je ressens. | 1 | 2 | 3 | 4 | 5 |
| 26 | Je m’égare dans mes pensées lorsque les autres parlent de leurs sentiments et de leurs besoins. | 1 | 2 | 3 | 4 | 5 |
| 27 | Il est inutile de s’interroger sur les intentions et les désirs des autres puisque nous dépendons tous de circonstances de vie. | 1 | 2 | 3 | 4 | 5 |
| 28 | L’une des choses les plus importantes que les enfants devraient apprendre est l’expression de leurs sentiments et de leurs désirs. | 1 | 2 | 3 | 4 | 5 |

**S4. Consortia and group authorship**

The following researchers contributed to this work as part of the Réseau Francophone des Thérapies Basées sur la mentalisation (RF-TBM) Consortium:

**Mario Speranza**

Paris-Saclay University, UVSQ, INSERM, Center for Epidemiology and Population Health Team “DevPsy”, Villejuif, France

University Department of Child and Adolescent Psychiatry, Versailles Hospital Center, Le Chesnay-Rocquencourt, France

**Paco Prada**

Consultation Liaison and Crisis Intervention, University Hospitals of Geneva, Switzerland

Department of Psychiatry, Geneva University Hospital, Geneva, Switzerland; Department of Medicine, University of Geneva, Geneva, Switzerland

**Pablo Cascone**

Division of Child and Adolescent Psychiatry, Department of Psychiatry, University Hospital of Lausanne and University of Lausanne, Lausanne, Switzerland

**Jalal Belmioud**

Office Médico-Pédagogique Research Unit, Department of Psychiatry, University of Geneva School of Medicine, Geneva, Switzerland
