# Supplementary material for: Validation of the Mentalization Scale (MentS) in francophone control and clinical samples
Source: PLoS One. 2025 Oct 28;20(10):e0332724. doi: 10.1371/journal.pone.0332724 (PMC12561985; doi:10.1371/journal.pone.0332724)
Supplement: Attachment — Submitted filename: Response to reviewers.docx [file pone.0332724.s002.docx]

PONE-D-25-06596

Validation of Mentalization Scale (Ment-S) in francophone control and clinical samples

PLOS ONE

**Rebuttal letter – Answer to the comments**

Please include the following items when submitting your revised manuscript:

- A rebuttal letter that responds to each point raised by the academic editor and reviewer(s). You should upload this letter as a separate file labeled 'Response to Reviewers'.
- A marked-up copy of your manuscript that highlights changes made to the original version. You should upload this as a separate file labeled 'Revised Manuscript with Track Changes'.
- An unmarked version of your revised paper without tracked changes. You should upload this as a separate file labeled 'Manuscript'.

Each of these items with mentioned labels have been uploaded.

Guidelines for resubmitting your figure files are available below the reviewer comments at the end of this letter.

Journal Requirements:

When submitting your revision, we need you to address these additional requirements.

1. Please ensure that your manuscript meets PLOS ONE's style requirements, including those for file naming. The PLOS ONE style templates can be found at <https://journals.plos.org/plosone/s/file?id=wjVg/PLOSOne_formatting_sample_main_body.pdf> and <https://journals.plos.org/plosone/s/file?id=ba62/PLOSOne_formatting_sample_title_authors_affiliations.pdf>

We have looked at the journal’s style requirements and have done modifications consequently to meet the journal’s style requirement for file naming.

2. We note that you have indicated that there are restrictions to data sharing for this study. For studies involving human research participant data or other sensitive data, we encourage authors to share de-identified or anonymized data. However, when data cannot be publicly shared for ethical reasons, we allow authors to make their data sets available upon request. For information on unacceptable data access restrictions, please see <http://journals.plos.org/plosone/s/data-availability#loc-unacceptable-data-access-restrictions>.

We hereby confirm that our data cannot be publicly shared based on the study’s ethics committee (Project-ID 2021-00694), but we do make it available upon request for a specific collaboration, which will entail a special permission granted by the local ethics committee.

Before we proceed with your manuscript, please address the following prompts:

a) If there are ethical or legal restrictions on sharing a de-identified data set, please explain them in detail (e.g., data contain potentially identifying or sensitive patient information, data are owned by a third-party organization, etc.) and who has imposed them (e.g., a Research Ethics Committee or Institutional Review Board, etc.). Please also provide contact information for a data access committee, ethics committee, or other institutional body to which data requests may be sent.

There are ethical and legal restrictions preventing the sharing of a de-identified dataset, as such sharing is not permitted under the ethics agreement approved by the cantonal ethics committee (Project-ID 2021-00694). Although authorization was granted to collect and analyze the data, sharing the dataset is prohibited due to the sensitive nature of the personal information involved, linked to the rules governing data collection locally in the hospital setting. The ethics committee prohibits the sharing of data. As a result, the dataset cannot be made publicly available, only upon request for a specific collaboration, which will entail a special permission granted by the local ethics committee.

Address of the cantonal authority on human medical research:

Commission cantonale d'éthique de la recherche (CCER)
Rue Adrien-Lachenal 8
1207 Genève
Phone: [+41 22 546 51 01](tel:+41225465101)
E-mail: [ccer@etat.ge.ch](mailto:ccer@etat.ge.ch)

b) If there are no restrictions, please upload the minimal anonymized data set necessary to replicate your study findings to a stable, public repository and provide us with the relevant URLs, DOIs, or accession numbers. Please see <http://www.bmj.com/content/340/bmj.c181.long> for guidelines on how to de-identify and prepare clinical data for publication. For a list of recommended repositories, please see <https://journals.plos.org/plosone/s/recommended-repositories>. You also have the option of uploading the data as Supporting Information files, but we would recommend depositing data directly to a data repository if possible.

Please update your Data Availability statement in the submission form accordingly.

There are restrictions as stated above. Therefore, we did not update the Data Availability statement. As it already stated in the original submission: *All relevant data are within the manuscript and its Supporting Information files*.

3. We note that your Data Availability Statement is currently as follows: All relevant data are within the manuscript and its Supporting Information files.

Please confirm at this time whether or not your submission contains all raw data required to replicate the results of your study. Authors must share the “minimal data set” for their submission. PLOS defines the minimal data set to consist of the data required to replicate all study findings reported in the article, as well as related metadata and methods (<https://journals.plos.org/plosone/s/data-availability#loc-minimal-data-set-definition>).

For example, authors should submit the following data:

- The values behind the means, standard deviations and other measures reported;
- The values used to build graphs;
- The points extracted from images for analysis.

Authors do not need to submit their entire data set if only a portion of the data was used in the reported study.

If your submission does not contain these data, please either upload them as Supporting Information files or deposit them to a stable, public repository and provide us with the relevant URLs, DOIs, or accession numbers. For a list of recommended repositories, please see <https://journals.plos.org/plosone/s/recommended-repositories>.

If there are ethical or legal restrictions on sharing a de-identified data set, please explain them in detail (e.g., data contain potentially sensitive information, data are owned by a third-party organization, etc.) and who has imposed them (e.g., an ethics committee). Please also provide contact information for a data access committee, ethics committee, or other institutional body to which data requests may be sent. If data are owned by a third party, please indicate how others may request data access.

There are ethical and legal restrictions on sharing a de-identified data set, as sharing data is prohibited under the current ethics agreement approved by the cantonal ethics committee. While authorization was granted to collect and analyze the data, sharing the dataset is not allowed due to the sensitive nature of the personal information involved. Consequently, as stated above, the dataset must remain confidential and cannot be shared.

Address of the cantonal authority on human medical research:

Commission cantonale d'éthique de la recherche (CCER)

Rue Adrien-Lachenal 8

1207 Genève

Phone: [+41 22 546 51 01](tel:+41225465101)
E-mail: [ccer@etat.ge.ch](mailto:ccer@etat.ge.ch)

4. Please amend your list of authors on the manuscript to ensure that each author is linked to an affiliation. Authors’ affiliations should reflect the institution where the work was done (if authors moved subsequently, you can also list the new affiliation stating “current affiliation:….” as necessary).

We have amended our list of authors to ensure that it reflects the institution where the work was done. Additionally, we have added Dr Eva Rüfenacht from Geneva University Hospitals who was also part of the data collection as well as the RFTBM consortium (Réseau Francophone de Thérapie Basée sur la Mentalisation) in the Supporting Information file. This addition exhaustively and more precisely reflects involvement in the work done and data collected.

5. We note you have included a table to which you do not refer in the text of your manuscript. Please ensure that you refer to Table 1 in your text; if accepted, production will need this reference to link the reader to the Table.

We thank you for your attention to this error and have made modifications accordingly.

6. We notice that your supplementary tables 5, and 6 are included in the manuscript file. Please remove them and upload them with the file type 'Supporting Information'. Please ensure that each Supporting Information file has a legend listed in the manuscript after the references list.

We thank you for your attention to this error and have made modifications accordingly.

[Note: HTML markup is below. Please do not edit.]

Reviewers' comments:

Reviewer's Responses to Questions

**Comments to the Author**

1. Is the manuscript technically sound, and do the data support the conclusions?

The manuscript must describe a technically sound piece of scientific research with data that supports the conclusions. Experiments must have been conducted rigorously, with appropriate controls, replication, and sample sizes. The conclusions must be drawn appropriately based on the data presented.

Reviewer #1: Partly

Reviewer #2: Yes

Reviewer #3: Partly

2. Has the statistical analysis been performed appropriately and rigorously?

Reviewer #1: Yes

Reviewer #2: Yes

Reviewer #3: Yes

3. Have the authors made all data underlying the findings in their manuscript fully available?

The [PLOS Data policy](http://track.editorialmanager.com/CL0/http:%2F%2Fwww.plosone.org%2Fstatic%2Fpolicies.action%23sharing/1/010f019696149a6e-88419235-78ef-43df-93eb-2d9eecead88b-000000/q6kDrncXY2tiYXCqY2ccA0F4UYT0DerNUhp-qqtHHGM=209) requires authors to make all data underlying the findings described in their manuscript fully available without restriction, with rare exception (please refer to the Data Availability Statement in the manuscript PDF file). The data should be provided as part of the manuscript or its supporting information, or deposited to a public repository. For example, in addition to summary statistics, the data points behind means, medians and variance measures should be available. If there are restrictions on publicly sharing data—e.g. participant privacy or use of data from a third party—those must be specified.

Reviewer #1: Yes

Reviewer #2: Yes

Reviewer #3: No

4. Is the manuscript presented in an intelligible fashion and written in standard English?

PLOS ONE does not copyedit accepted manuscripts, so the language in submitted articles must be clear, correct, and unambiguous. Any typographical or grammatical errors should be corrected at revision, so please note any specific errors here.

Reviewer #1: Yes

Reviewer #2: Yes

Reviewer #3: No

5. Review Comments to the Author

Please use the space provided to explain your answers to the questions above. You may also include additional comments for the author, including concerns about dual publication, research ethics, or publication ethics. (Please upload your review as an attachment if it exceeds 20,000 characters)

**Reviewer #1:**

- This study evaluated the psychometric properties of the French version of the Mentalization Scale (MentS) in both community and clinical samples. A total of 711 participants, including individuals with borderline personality disorder (BPD), ADHD, and co-occurring BPD and ADHD, completed the scale. Confirmatory factor analysis supported a 27-item, three-factor structure (mentalizing self, mentalizing others, and motivation to mentalize) as optimal for both groups. According to the Authors, the French version of the MentS is suitable for research and clinical use.
  The topic of the paper is both timely and interesting. However, there are several important points that require attention. I recommend a thorough and very careful revision of the manuscript to address these issues and strengthen the overall quality of the work.

In the Introduction, the Authors present the RFS scale in a somewhat cursory manner. It would have been beneficial to provide a more comprehensive overview of the RFS, including discussion of related instruments such as the RFQ-8. Notably, the authors do not address several critical issues—frequently highlighted in the literature—regarding the challenges of measuring mentalization with the RFS. As a result, the transition to the MentS scale feels abrupt and insufficiently justified. Furthermore, it is important to acknowledge that, both before and after the development of the MentS, other instruments for assessing mentalization have been introduced. Including references to these alternative measures would have strengthened the context and provided a more balanced perspective.

In the Introduction (Lines 80–92), the authors assert that Fonagy and Luyten have addressed and resolved the methodological limitations outlined earlier (Lines 77–79). They also cite encouraging data supporting the francophone version of the RFQ. However, the subsequent transition to introducing the Mentalization Scale (MentS) feels abrupt and insufficiently justified. While the MentS is indeed a valuable tool for multidimensional mentalization assessment, the authors do not adequately articulate its comparative advantages over existing measures. A more thorough discussion is needed to clarify why the MentS was selected over alternative instruments, particularly in light of its strengths (e.g., multidimensional structure) and potential limitations. Highlighting how the MentS addresses gaps left by other tools would provide a stronger rationale for its use and enhance the coherence of this section.

We thank the reviewer for this helpful and constructive comment.

The line numbers mentioned in our responses refer to the clean version of the manuscript.

In the revised manuscript, we expanded the paragraph originally spanning lines 78 to 90 to provide a more comprehensive and fine-grained presentation of the Reflective Functioning Scale (RFS), also mentioning the well-documented limitations in assessing mentalization (lines 74 to 82).
This section is now complemented by a similarly concise yet thorough overview of the Reflective Functioning Questionnaire (RFQ), focusing specifically on the 8-item version (lines 90 to 97)

We then briefly introduce earlier self-report measures developed prior to the MentS, namely the Mentalization Questionnaire (2012) and the Mentalized Affectivity Scale (MAS) (2017), as well as the mention of other more recent self-report developed tools since the MentS (lines 97 to 108).

Finally, at the end of this revised section, once all relevant tools have been introduced and the MentS is appropriately situated among them, we inserted a new paragraph assessing elements specific to the MentS in comparison to existing instruments (lines 109 to 113).

- In my view, the description of the MentS in the manuscript is insufficient and does not offer readers a clear understanding of the instrument. The structure and dimensions of the MentS are not adequately defined, while greater emphasis is placed on the interpretation of scores. Providing a more detailed explanation of the scale’s underlying structure and its specific dimensions would significantly enhance the reader’s comprehension and better contextualize the meaning and relevance of the reported scores.

In the revised version of the manuscript, we have added elements to provide a better understanding of the MentS instrument. Specifically, we now include a clear explanation of each dimensions composing the structure of the MentS, as well as example items for each subscale. We further make explicit the number of items per subscale (lines 113 to 129) and provide the full French-translated instrument as supplementary information to the article.

- I found the section of the Introduction addressing convergent validity (lines 128 to 134) to be somewhat confusing, possibly due to an oversimplification of the authors’ argument. The formulation of multiple hypotheses in the latter part of the introduction is particularly perplexing, as several of these hypotheses, especially the one regarding a negative correlation between the MentS-S scale and self-reported childhood trauma, are not logically substantiated within the text.

We thank the reviewer for this constructive observation. We acknowledge that the section on convergent validity may have appeared confusing due to an overly condensed presentation of our rationale.
In the revised manuscript we have clarified and expanded this section. We articulated the arguments in favor of the validation choices and oriented the reader to the relevant literature sustaining these arguments. Following these modifications, this section more explicitly renders the theoretical and empirical basis for each hypothesis (lines 155 to 178). Specifically, we cite the studies reporting a negative correlation between the MentS-S subscale and self-reported childhood trauma (lines 179 to 186).

- Additionally, I am concerned by the decision to assess childhood trauma using a self-report measure, as this approach raises questions about the

Regarding the use of a self-report measure for assessing childhood trauma, we had acknowledged the limitations inherent to retrospective self-report instruments. Following the reviewer comment, we have expanded the limitations section of the manuscript to explicitly discuss the potential biases associated with self-reported trauma data and to emphasize the need for future studies to incorporate complementary assessment methods (lines 570 to 579).

- A seemingly minor point concerns the way references are cited within the text. While I understand that the authors may have chosen this citation format to simplify the preparation of the reference list, it does not conform to any recognized citation styles typically used in scientific writing. Adhering to a standard citation style is important for clarity and consistency throughout the manuscript.

Thank you for this helpful comment. Indeed, we noticed that narrative in-text citations were not consistent with the Vancouver style. This has now been corrected accordingly throughout the manuscript. The overall citation format follows the Vancouver style, as required by the journal and implemented via EndNote.

- Method
  In describing the sample, the authors state that they recruited participants for the control group via the Prolific website. Based on my understanding, Prolific is a platform that compensates research participants for their time and contributions. While this approach facilitates rapid and diverse recruitment, it does raise the question of whether such a method increases the likelihood of enrolling so-called **“**professional participants”—individuals who frequently participate in online studies for compensation. It is still possible for samples to include individuals with substantial prior experience in online research, which may influence their responses or introduce certain biases. While Prolific offers valuable tools to mitigate the risk of recruiting predominantly “professional participants,” researchers should remain mindful of this potential limitation and consider reporting the level of participant experience in their sample description.

We thank the reviewer for raising this important point which we now mention in our limitation’s section (lines 566 to 568).

The measures taken to strengthen the quality of the data collection include monitoring the time taken to complete the self-reports, and the inclusion of attention check items. Regarding these control items, participants were excluded if they gave incorrect answers to more than two bogus items, or if their response time was faster than the mean of the response times measured on the whole sample (difference – 2 s.d.). Also, participants who gave an incorrect answer to only one bogus item could be excluded if their total response time was faster than the mean by at least – 1 s.d. . As a result of the combination of these rules, N = 70 participants were excluded. We have added this information in the revised manuscript (lines 209 to 216).

- Including a brief description of the MentS and reporting Cronbach's alpha values at the end of the Participants paragraph is unusual and disrupts the logical flow of the manuscript. The Participants section should focus on describing the sample's characteristics and recruitment methods, as well as any relevant demographic information. Details about the instruments used—including their structure, dimensions, and psychometric properties such as reliability (e.g., Cronbach's alpha)—are more appropriately placed in the Materials or Measures section.
  In summary, the inclusion of the MentS description and its Cronbach's alpha values in the Participants paragraph is misplaced. This information would be more appropriately presented in the section dedicated to describing the study's materials or measures, where readers expect to find details about the instruments and their psychometric properties.

Consistently with this comment, we have moved the relevant passage to the Measures section to improve the organization and clarity of the manuscript (lines 250 to 254).

- Additional measures
  The section describing additional measures administered alongside the MentS lacks clarity regarding the rationale for selecting these instruments. It is important for the authors to explain why each measure was included and how it relates to the study’s objectives or hypotheses. Without this context, readers may find it difficult to understand the relevance and contribution of these additional assessments.

As mentioned above following one of the reviewer’s previous comments, the rationale for selecting these instruments has been expanded in the introduction section (lines 155 to 186).

- Furthermore, the information about the translation into French, which appears at the end of this paragraph, seems out of place. Details about translation procedures are typically presented in a dedicated section on instrument adaptation or within the description of the specific measure being translated. Placing this information in the "Additional Measures" section disrupts the logical flow and organization of the manuscript.
  To improve clarity and coherence, the authors should clearly justify the inclusion of each additional measure and relocate the translation details to a more appropriate section of the manuscript.

Consistently with this comment, we have moved the information regarding the French translation to the measures section for improved clarity (lines 254 to 257).

- As a minor point, it should be noted that the authors used the RFQ-8, the short form of the Reflective Functioning Questionnaire, rather than the longer version of the instrument.

Consistently with this comment, we have specified the use of the 8-item of the RFQ clearly both in the introduction and in the description of the instruments (line 281).

- Statistical analysis
  Based on the results of the confirmatory factor analysis (CFA) conducted on both the control and clinical samples, the authors decided to eliminate item 25 from their version of the MentS. After removing item 25, the authors used this abbreviated version of the MentS for subsequent reliability analyses. Then they computed the associations between the measures of interest in both samples. The lack of clarity regarding the MentS scoring system in the manuscript creates significant ambiguity, particularly in interpreting the meaning of positive or negative values.

In the revised manuscript, we have added a paragraph explicitly describing the MentS scoring procedure to improve clarity for the reader, which we hope increases overall clarity (lines 424 to 428).

Additionally, we now mention the interpretation behind greater and diminished values of the MentS scores in the Measures section (line 248).

- The fact that the authors used the Jamovi software for statistical analyses should have been stated at the beginning of the paragraph**,** not at the end of the section dedicated to the CFA.

The modification was performed accordingly, thank you (lines 297-298).

- Discussion
  In this section of the manuscript, the authors' extensive focus on findings derived from the Childhood Trauma Questionnaire (CTQ) short form—a 28-item self-report measure—warrants further scrutiny. Although the CTQ is a validated instrument, its brevity and reliance on retrospective self-reporting raise concerns about its ability to comprehensively assess complex and multifaceted experiences of childhood maltreatment. The CTQ evaluates five subscales (physical, emotional, and sexual abuse, along with emotional and physical neglect), yet such a condensed format may lack the depth needed to capture the nuances of traumatic experiences, including contextual factors, chronicity, and subjective impact. Given these limitations, the heavy emphasis on CTQ-based results in the discussion may inadvertently oversimplify the interpretation of childhood trauma's role in the study's outcomes.
  It is worth to note that self-report measures are vulnerable to recall bias, social desirability bias, and underreporting, especially for stigmatized experiences like abuse. Participants may consciously or unconsciously minimize or deny traumatic events. The CTQ quantifies maltreatment severity but provides no qualitative insights into the lived experience of trauma. Critical factors such as developmental timing, relational dynamics, and coping mechanisms remain unaddressed.
  Finally, the CTQ’s subscales (physical/emotional/sexual abuse, physical/emotional neglect) show high intercorrelations, making it difficult to isolate specific trauma types. This overlap complicates interpretations of how distinct maltreatment experiences relate to outcomes like mentalization deficits.
- The brief mention of the study’s limitations in the final section of the discussion is inadequate. A thorough and transparent discussion of limitations is essential for contextualizing the findings, acknowledging potential biases, and guiding future research. Simply referencing these issues without elaboration does not provide readers with a clear understanding of how the study’s design, measures, or sample characteristics may have influenced the results. Expanding this section to address specific methodological constraints, such as the reliance on self-report measures, sample representativeness, or the generalizability of the findings, would strengthen the manuscript and enhance its scientific rigor.

In response to this comment, we have now expanded the limitations’ section to more transparently address the methodological constraints. By acknowledging these limitations explicitly, we aim to provide a more nuanced interpretation of our findings and a clearer direction for future research (lines 566 to 582).
Indeed, whilst the Childhood Trauma Questionnaire (CTQ) short form is a widely used and validated measure for assessing childhood maltreatment, we fully acknowledge its inherent limitations and have revised the discussion accordingly as stated above.

In agreement with the Reviewer, we attempted to nuance the “heavy emphasis on CTQ-based results” by adapting the discussion towards the links between childhood trauma and the development of psychopathology. (lines 521 to 531)

We wish to thank the reviewer for this set of comments which we feel have helped us make our manuscript clearer and more to the point.

**Reviewer #2:**

- Although the authors justify model refinements (e.g., residual correlations and removal of item 25), a brief expanded discussion on clinical interpretation and usability of the shortened 27-item scale would benefit readers. Implications for practitioners using this tool in diagnostic or therapeutic settings could be elaborated—especially given the growing interest in mentalization in clinical psychology.

We thank the reviewer for this helpful suggestion. In the revised manuscript, we expanded the discussion (lines 549 to 564) in accord with the review’s suggestion.

- A few language polishing points (minor grammar or flow) may help improve readability, though these are not major.

We thank the reviewer for this remark. Before final submission, and for the revised version of the manuscript, the text has been carefully reviewed and to improve language, grammar, and flow.

**Reviewer #3:**

- The present study explores the psychometric properties of the francophone translation of the MentS, performing a confirmatory factor analysis and evaluating test-retest reliability.
  The study is sound. However, I would like to draw your attention to some areas for improvement.
- First of all, the acronym MENT-S appears in the title, while MentS is used throughout the manuscript. I recommend choosing one version and using it consistently.

We thank the reviewer for this observation. In the revised version of the manuscript, we were careful to ensure consistency throughout the manuscript while choosing the “MentS” acronym.

- In general, I suggest reviewing spelling according to a consistent language style (British or American English, e.g., behavior / behaviour).
  I also recommend that the authors revise the writing and the English language throughout the manuscript, as some sentences are difficult to understand or seem incomplete, and there are a few mistakes. Below are some examples:
  - Line 22 ("it's" should be "its"?)
  - Lines 33–34 (revise sentence structure)
  - Line 48 ("may more likely to report increased")
  - Line 58 ("The centrality of mentalizing in human" → "mentalization")
  - Lines 150–151 (combine the two sentences)
  - Lines 161–162 (combine the two sentences)
  - Line 165 ("sample composed of" → "sample was composed of")
  - Line 252 ("analysis were" → "analysis was")
  - Lines 308–309 (split the sentence using a full stop → ". Results ...")
  - Line 352 (some values are reported as r=xxx, others as r = xxx. Decide on spacing and ensure consistency)

We thank the reviewer for this remark. For the revised version of the manuscript, we had the text carefully reviewed to improve language, grammar, and flow. Additionally, we were careful to further ensure consistency with reported values as mentioned in your comment.

- I also recommend avoiding the use of asterisks in the text to indicate p-values (it is acceptable in tables, but in the text, it is always preferable to report values fully, e.g., p < 0.001).

Furthermore, in table- section "note", when acronyms are explained, the initial letters of the words should be capitalized (e.g., ADHD = Attention Deficit Hyperactivity Disorder; RMSEA = Root Mean Square Error of Approximation).

Abstract: I suggest reporting the test-retest reliability values in parentheses and naming at least the constructs that were assessed when referring to "additional measures" (line 40).

Consistently with these comments, we revised the manuscript accordingly.

(Modification of abstract in lines 37 to 40, and removal of asterisks from text in lines 453 to 461 & 464 to 473)

- Methods:
  - Lines 173–175 would be more appropriate in the Results section.
  - I would move the following line (inclusion criteria at the end of line 169).
  - Before describing the various measurement instruments, insert a subheading titled

We revised the manuscript accordingly.

The lines 173–175 were moved to the Results section (lines 387-388).

The inclusion criteria were moved (lines 227 to 230 and a subheading was added before the description of the measurement instruments (line 242).

- Measures:
  -The section on missing data should be placed under Data Analysis and should specify whether any missing data were present. Also, provide a reference for the rule “If less than 30% of item values were missing”.

We revised the manuscript accordingly and specified the presence of missing data.

Additionally, we now added a reference to the rule (Line 302).

- - Line 291–292: specify why the non-parametric Spearman coefficient was used (were the variables not normally distributed?). Additionally, there is a lack of detail regarding outliers, multicollinearity, heteroscedasticity, etc.

To make things clearer regarding the distribution of the data, we added the following paragraph in the method section (lines 346 to 353):

“Normality of score distributions for all MentS subscales and total scores was evaluated through skewness, kurtosis, and the Shapiro-Wilk test. These indices were computed separately for the clinical and control samples. Following common guidelines, distributions were considered approximately normal when skewness and kurtosis values fell within the ±2 range (78), and when the Shapiro-Wilk test was non-significant (p > .05) (79). Shapiro-Wilk tests indicated no violation of normality for the total scores in either group. All subscales were normally distributed in the control group, whereas in the clinical group, the Others and Self subscales significantly deviated from normality, while the Motivation subscale did not.”

Additionally, to make things clearer regarding the outliers and multicollinearity, we added the following (lines 307 – 322):

“Visual inspection of histograms, and boxplots was also performed to assess the shape of distributions and detect potential outliers. Extreme values were identified in the control group but not in the clinical sample. Specifically, one extreme low value was observed on the self-mentalizing subscale (MentS-Self), while no outliers were detected on the MentS-Others or MentS-Motivation subscales. On the MentS total scale, six extreme values were found: three on the lower end and three on the higher end. All data points, including these extreme cases, were retained in the analyses, as they were considered to reflect meaningful individual variation rather than error. As a result, nonparametric Spearman’s rho correlations were computed to examine the associations among the MentS subscales in the control group to assess potential multicollinearity. A moderate positive correlation was found between MentS-Others and MentS-Motivation (ρ = .703, p < .001), indicating substantial overlap between these two dimensions. Weaker but significant correlations were also observed between MentS-Self and MentS-Others (ρ = .175, p = .002), and between MentS-Self and MentS-Motivation (ρ = .120, p = .037). These results suggest that while the three subscales are related, they capture distinct facets of mentalizing. All correlations fell within acceptable ranges (71), indicating no significant concerns regarding multicollinearity.”

Finally, heteroscedasticity is now addressed in the following lines (327 – 329): “The WLSMV estimator is well suited for ordinal data and provides robust estimates in the presence of non-normality and potential heteroscedasticity.”

- While the general approach for statistical analyses is sound and report a comprehensive set of fit indices was reported, several statistical concerns and methodological considerations should be addressed to enhance the rigor and interpretability of the results.The use of ML estimation may not be optimal given that questionnaire data are typically ordinal in nature. ML assumes multivariate normality and continuous data, which is often violated in Likert-type scales. A more appropriate estimation method would be Weighted Least Squares Mean and Variance adjusted (WLSMV), which is robust for ordinal variables and commonly recommended in such contexts.

We thank the reviewer for this relevant and insightful observation. We acknowledge that the initial analyses used maximum likelihood (ML) estimation, which assumes continuous and normally distributed data and is hence a mistake in our statistical approach – we therefore reconducted all confirmatory factorial analysis with the correct estimator.

These analyses were performed using the SEMlj module in Jamovi, through syntax-based specification. The WLSMV-based CFA analyses now replace the original ML-based analyses in the manuscript to ensure a more statistically appropriate and robust approach.

Modifications of results can be found from lines 394 to 419.

The method section has been modified accordingly (lines 325 to 344).

- The final model incorporates 24 correlated residuals, which is a substantial number relative to the total number of items (28). While the justification provided (i.e., similar item wording) is acknowledged, such an extensive modification raises concerns about overfitting and may artificially inflate model fit. Correlated errors should be added sparingly and only when strong theoretical justification is present. I read that you have brought this observation within limitation of the study.

In response to the reviewer’s comments and previous modification suggestions, we re-estimated all CFA models using WLSMV estimation instead of ML.

This led to a considerable simplification of the model: only three theoretically justified correlated residuals remain in the final solution (line 400). We believe this revised structure addresses the reviewer’s concern about potential overfitting.

We are grateful for the reviewer’s guidance on this point, which directly contributed to a more statistically rigorous and parsimonious model. Given the minimal number of residual covariances retained, we no longer view this as a limitation.

- After introducing model modifications and removing item 25, it would be important to statistically compare the models (e.g., using Chi-square difference tests or ΔCFI/ΔRMSEA) to support the decision to retain the revised structure.

We thank the reviewer for this valuable and precise suggestion. In the revised manuscript, we conducted statistical comparisons between the three confirmatory factor analysis (CFA) models tested, to justify the progression from the original structure to the final, revised model (lines 421 to 423).

- Additionally**,** cross-validation using split samples or bootstrapping could help assess the stability of the modified model.

In response to this comment, we performed a between-sample cross-validation to assess the stability and generalizability of the revised CFA model, rather than simply conducting separate confirmatory factor analyses (CFAs) in both samples.

Specifically, we first estimated the revised model in the control group using WLSMV estimation. The yielded best-fitting model included a reduced number of theoretically justified correlated residuals and excluded item 25 based on its poor performance. The model demonstrated excellent fit in the control group (CFI = 0.954, TLI = 0.949, RMSEA = 0.071, SRMR = 0.079).

We then cross-validated this same model, without any structural modifications, in the independent clinical sample. The model yielded acceptable fit indices in this group as well (CFI = 0.902, TLI = 0.891, RMSEA = 0.069, SRMR = 0.077). Notably, RMSEA and SRMR values were highly consistent across both groups.

Although a slight reduction in CFI and TLI was observed in the clinical group, these indices remained within acceptable thresholds. Taken together, these findings support the stability and replicability of the final CFA model across independent samples, in line with the reviewer’s recommendation.

These modifications are found in the article in the method (lines 329 to 335) and results (lines 394 to 418) section.

- you briefly mention the use of Spearman’s rho due to non-normal distributions but, as previously mentioned, other essential assumptions such as outliers, multicollinearity, and heteroscedasticity are not addressed. Clarification on these points would strengthen the statistical validity of the analyses.

We thank the reviewer for this helpful remark. In the revised manuscript, we provided additional information as stated above in your previous point concerning the similar observation.

- Given the large number of residual correlations (24 pairs) needed to improve model fit in the confirmatory factor analysis, I wonder whether you considered using an Exploratory Structural Equation Modeling (ESEM) approach. ESEM could have allowed for more flexibility in modeling item cross-loadings without relying on post-hoc correlated error terms and might have provided a better representation of the underlying structure. Including a rationale for not choosing this approach, or a brief discussion of its potential relevance, would strengthen the methodological justification of the CFA strategy adopted.

We fully acknowledge that Exploratory Structural Equation Modeling (ESEM) provides greater flexibility by allowing for cross-loadings and may, in some cases, reduce the need to specify numerous residual correlations. However, in the present study, we chose to apply a strictly confirmatory factor analysis (CFA) approach. This decision was supported by ten prior validation studies of the MentS, which have consistently replicated the hypothesized three-factor structure across diverse populations. This body of evidence provided a strong empirical and theoretical basis for evaluating the model within a confirmatory framework.

Considering the reviewer’s suggestion, we emphasize that several steps were taken to strengthen the confirmatory analysis: (1) the WLSMV estimator was used to handle ordinal data appropriately; (2) the number of residual correlations was reduced; and (3) the model was cross validated across two independent samples (control and clinical). The model demonstrated satisfactory fit in both groups, supporting its stability and generalizability.

Taken together, we believe that the confirmatory framework has been rigorously and appropriately applied, and that the additional exploratory modeling (e.g., ESEM) is not warranted at this stage.

6. PLOS authors have the option to publish the peer review history of their article ([what does this mean?](https://track.editorialmanager.com/CL0/https:%2F%2Fjournals.plos.org%2Fplosone%2Fs%2Feditorial-and-peer-review-process%23loc-peer-review-history/1/010f019696149a6e-88419235-78ef-43df-93eb-2d9eecead88b-000000/ZcdJX-dXrAynFEdzL8lHBeJNCGUmt5_ARHsKmieQMps=209)). If published, this will include your full peer review and any attached files.


If you choose “no”, your identity will remain anonymous but your review may still be made public.


**Do you want your identity to be public for this peer review?** For information about this choice, including consent withdrawal, please see our [Privacy Policy](https://track.editorialmanager.com/CL0/https:%2F%2Fwww.plos.org%2Fprivacy-policy/1/010f019696149a6e-88419235-78ef-43df-93eb-2d9eecead88b-000000/SDErOT-CMDlM0aOiao_xSbADwbbCNVdn-lRbX7_Nm14=209).

Reviewer #1: No

Reviewer #2: No

Reviewer #3: No

[NOTE: If reviewer comments were submitted as an attachment file, they will be attached to this email and accessible via the submission site. Please log into your account, locate the manuscript record, and check for the action link "View Attachments". If this link does not appear, there are no attachment files.]

While revising your submission, please upload your figure files to the Preflight Analysis and Conversion Engine (PACE) digital diagnostic tool, [https://pacev2.apexcovantage.com/](https://track.editorialmanager.com/CL0/https:%2F%2Fpacev2.apexcovantage.com%2F/1/010f019696149a6e-88419235-78ef-43df-93eb-2d9eecead88b-000000/bjEr_Nuc-WaLi5FQMcv81qMc4kzeMRCh9WP5ke2JHFc=209). PACE helps ensure that figures meet PLOS requirements. To use PACE, you must first register as a user. Registration is free. Then, login and navigate to the UPLOAD tab, where you will find detailed instructions on how to use the tool. If you encounter any issues or have any questions when using PACE, please email PLOS at [figures@plos.org](mailto:figures@plos.org). Please note that Supporting Information files do not need this step.
